# Analysis of induced abortion-related complications in women admitted to referral health facilities in Kinshasa, Democratic Republic of the Congo

**DOI:** 10.1371/journal.pone.0203186

**Published:** 2018-08-30

**Authors:** Daniel Katuashi Ishoso, Antoinette Kitoto Tshefu, Yves Coppieters

**Affiliations:** 1 Community Health Department, Kinshasa School of Public Health, University of Kinshasa, Kinshasa1, Democratic Republic of Congo; 2 Research Centre "Policies and Health Systems—International Health", School of Public Health, Université libre de Bruxelles (ULB), Brussels, Belgium; Guttmacher Institute, UNITED STATES

## Abstract

**Background:**

Due to a lack of relevant data on induced abortions in the Democratic Republic of the Congo (DRC), as well as the persistence of maternal deaths in the country, this study aims to analyze the extent and characteristics of induced abortion-related complications in women who were admitted to referral health facilities in Kinshasa, including the duration of hospitalization, the mortality rate due to induced abortion complications and their characteristics, and the deaths that occurred after two days of hospitalization.

**Methods:**

This is a cross-sectional study on 843 obstetric and gynecological patients who were admitted as emergency cases to five referral health facilities in Kinshasa during 2014. These facilities were selected as being representative of five types of districts in Kinshasa, according to their cultural, socioeconomic, and infrastructural characteristics. Patient data were collected from patient records and analyzed.

**Results:**

From the 843 patients admitted to receive obstetric and gynecological emergency care services in 2014 at the health facilities surveyed, 14.7% (95% CI: 12.4–17.3%) had complications due to induced abortion. These complications were significantly diagnosed in adolescents (p = 0.003) and in single, separated, divorced, or widowed women (p = 0.03). The median duration of hospitalization was nine days, and this period of time was significantly longer for the patients who underwent surgery for pelvic peritonitis due to uterine perforation compared with the patients who underwent Caesarean section/hysterectomy. Furthermore, it was significantly longer for the patients who were treated for other induced-abortion related complications compared with patients treated for spontaneous abortion. The mortality rate related to induced abortions was 5.6% (95% CI: 2.3–11.3%), with an increase in risk of death in the presence of a postabortive pelvic peritonitis-type complication; 42.9% of deaths occurred after two days of hospitalization.

**Conclusion:**

The complications of induced abortions are a major public health problem due to their frequency among patients admitted to Kinshasa's referral health facilities, their mortality, and their poor medical management. Therefore, there is a need to understand the reason for its poor medical management in order to provide an adequate intervention program.

## Background

Approximately 25 million unsafe abortions are estimated to take place worldwide each year [1], the majority of which occur in developing countries where approximately 7 million women are admitted to hospitals every year as a result of complications [2].

Complications from unsafe abortions account for approximately 7.9% (4.7–13.2%) of maternal deaths [3]. In absolute terms, approximately 193,000 maternal deaths occur each year worldwide; 192,000 of these maternal deaths occur in developing countries, and sub-Saharan Africa has the largest share, with 125,000. In addition to maternal deaths, high morbidity can be observed long-term, including conditions such as premature births, psychological sequelae, infertility or subfertility, chronic pelvic pain, ectopic pregnancy, and spontaneous abortions [4, 5, 6, 7].

In the Democratic Republic of the Congo, the prevalence of modern contraception is low at 8% [8]. This likely explains the high rate of 147 unwanted pregnancies per 1000 women of childbearing age and the high rate of 57 unsafe abortions per 1000 women of childbearing age [9]. Although the provision of maternal care (antenatal care, assisted delivery supervised by trained personnel, etc.) has improved considerably in the DRC, the rate of maternal mortality is still high with 846 deaths per 100,000 live births [8]. In addition, the number of induced abortion-related complications, as well as deaths related to them, may be high and contribute significantly to the overall number of observed deaths. As these data are unavailable, this study proposes to analyze the cases of complications caused by induced abortions in patients who were admitted to referral health facilities in Kinshasa, the capital city of the DRC. Specifically, the objectives of this study are (i) to determine the proportion of induced abortion-related complications in women admitted to the obstetric and gynecological emergency unit of referral health facilities in Kinshasa, as well as their demographic and socioeconomic characteristics; (ii) to measure the duration of hospitalization of the patients; (iii) to determine the mortality rate due to induced abortion complications, as well as their characteristics; and (iv) to determine the proportion of deaths that occur after two days of hospitalization (conventionally, in the DRC, cause of death is evaluated at the health facility).

## Methods

### Study design

In April 2015, a cross-sectional study was carried out at the referral health facilities in Kinshasa. Data were collected retrospectively from 1^st^ January 2014 to 31^st^ December 2014.

### Study population

The study population included patients admitted to the obstetric and gynecological emergency units of referral health facilities in Kinshasa from the 1^st^ of January 2014 to the 31^st^ of December 2014.

### Sampling

A probabilistic sample by stratum was performed. Initially, the five types of neighborhoods in Kinshasa were considered, differentiated by their cultural, socioeconomic, and infrastructural characteristics [10]. For each type of neighborhood, one health zone was randomly drawn, and for each health zone, one secondary referral health facility was drawn.

All patients admitted to the obstetric and gynecological emergency units of Kinshasa's secondary referral health facilities from 1 January 2014 to 31 December 2014 were included.

The sample size was computed using the following formula:

$$n\geq \frac{{Z\alpha }^{2}.p.q}{d²}$$

where ‘p’ represents the proportion of induced abortion-related complications in women admitted to the obstetric and gynecological emergency unit (we used the proportion of 50% to have the highest minimum size); q represents the equation (1-p); z represents the value of the standard normal distribution coefficient corresponding to a significance level of alpha of 0.05 (1.96); and d represents the precision degree that we assumed to be at 5%. The minimal size computed was 384 patients.

### Data collection, information sources, and variables

Data were collected by four nurses and one physician using a standardized investigation sheet. Information required to complete the investigation sheet was obtained from obstetric and gynecological emergency and medical records.

The following variables were collected: demographic and social characteristics of patients (age, marital status, occupation), clinical characteristics of patients (temperature, parity, previous abortion, surgical history, clinical diagnosis, the results of laboratory analysis and ultrasound, definitive diagnostic, treatment, length of hospital stay, outcome) and healthcare system variables (waiting time).

Regarding the distinction between induced abortions and spontaneous abortions, the diagnosis of health care providers was guided by patient self-report.

As for the variable of age, the group of adolescents consisted of any subject aged 19 years or younger, which was in agreement with the UNICEF, UNAIDS, and WHO definitions.

### Data processing and statistical analysis

Data were entered into an Epi Info software program, version 3.5.4, exported to Microsoft Excel, and analyzed using Statistical Package for the Social Sciences (SPSS) version 20, IBM. Descriptive statistics were used to summarize the characteristics of the study population. Continuous variables were reported as follows: the mean with standard deviation for patient’s age, and the median with interquartile range for length of hospital stay.

The Mann-Whitney test was used to compare the median length of hospitalization among the different groups of dichotomous variables examined. Categorical variables were reported using frequency and percentage, and groups were compared using the χ2 test. The odds ratio (OR), with a corresponding 95% confidence interval, was reported to quantify the strength of association. P-values of less than 0.05 were considered significant.

### Ethical considerations

The National Committee of Ethics of the Kinshasa School of Public Health (NCE-KSPH) approved the present study. Authorization was also provided by health and politico-administrative authorities. Consent was obtained from supervisors at the selected medical facilities prior to the collection of data. The NCE-KSPH waived the need for consent of the participants because data were drawn from the medical records of patients who had either died or had been discharged from the hospital, and they insisted on the anonymity of patients in the collection and analysis of data. Anonymity was maintained during the collection of data, and the survey forms of data collected were safeguarded.

## Results

### Frequency of cases of induced abortion-related complications admitted to referral health facilities

In 2014, eight hundred and forty-three patients were admitted to obstetric and gynecological emergency units at the health facilities surveyed, of which 14.7% (95% CI: 12.4–17.3%) had complications due to induced abortion (Table 1).

**Table 1:** Frequency of cases of induced abortion-related complications.

Patients admitted n = 843	n’ (%)	CI 95%
Induced abortion-related complications cases		
Yes	124 (14.7)	12.4 to 17.3
No	719 (85.3)	82.7 to 87.6
Total	843 (100)	

n = number of subjects in the sample; n’ = number of subjects in the sample subgroups

### Characteristics of induced abortion-related complications

The mean age of the patient population was 29 years (± 7 years), and the majority of the patients were married or cohabiting, multiparous, and without a history of induced abortion. The adolescents had a significantly a higher risk to experience an induced abortion complication than adults (p = 0.003), and the single, separated, divorced, or widowed women had significantly higher risk than married women (p = 0.03).

Six and a half percent of the cases of induced abortion complications were admitted for postabortion pelviperitonitis (serious form of complication), and the remaining were admitted for infection or hemorrhage (Tables 2 and 3).

**Table 2:** Descriptive analysis.

Variables	n’ (%)	Average (SD)
**Patients’ place of residence (n = 843)**	** **	
Semirural areas	195 (23.1)	
Areas outside the city	214 (25.4)	
Areas in planned cities	170 (20.2)	
Areas in old cities	64 (7.6)	
Residential areas	136 (16.1)	
Total	843 (100)	
**Adolescence (years) (n = 808)**	** **	**29 (7)**
Yes	85 (10.5)	
No	723 (89.5)	
Total	808 (100)	
**Marital status (n = 127)**		
Married/cohabiting	111 (87.4)	
Single/separated/divorced/widowed	16 (12.6)	
Total	127 (100)	
**Patient parity (n = 430)**	** **	
Nulliparous	165 (38.4)	
Primiparous	91 (21.2)	
Multiparous	174 (40.5)	
Total	430 100)	
**Patient abortion history (n = 429)**		
No abortion history	283 (66.0)	
One or several abortions	146 (34.0)	
Total	429 (100)	
**Type of complications of induced abortion (n = 124)**		** **
Postabortion pelviperitonitis	8 (6.5)	
Others (infection, hemorrhage)	116 (93.5)	
Total	124 (100)	
**Types of cases requiring surgery through the abdomen (n = 599)**		
Postabortion pelviperitonitis	8 (1.3)	
Other diagnostics	591 (98.7)	
Total	599 (100)	
**Types of cases not requiring surgery through the abdomen (n = 224)**		
Other complications of induced abortion	116 (47.5)	
Spontaneous abortions	128 (52.5)	
Total	224 (100)	

n = number of subjects in the sample; n’ = number of subjects in the sample subgroups; SD = Standard Deviation

**Table 3:** Demographic, social and clinical characteristics of the women admitted for induced abortion-related complications.

Variables	Percentage (%) of cases: complication of induced abortion	OR (CI95%)	P
**Adolescence** (≤ to 19 yrs)	** **	** **	**0.003**
Yes (n’ = 85)	24.7	2.2 (1.3 to 3.8)	
No (n’ = 723)	12.9	1	
**Patients’ areas of residence**			**0.2**
Semirural areas (n’ = 195)	19.0	1.9 (0.8 to 4.8)	
Residential areas (n’ = 136)	11.8	1.1 (0.4 to 2.9)	
Areas outside the city (n’ = 214)	14.0	1.3 (0.6 to 3.4)	
Areas in planned cities (n’ = 170)	17.1	1.7 (0.7 to 4.3)	
Areas in old cities (n’ = 64)	10.9	1	
**Patient Marital Status**			**0.03**
Single/separated/divorced/widowed (n’ = 16)	25.0	4.8 (1.1 to 19.4)	
Married/cohabiting (n’ = 111)	6.3	1	
**Patient parity**			**0.8**
Nulliparous (n’ = 165)	10.3	1.6 (0.6 to 2.6)	
Primiparous (n’ = 91)	9.9	1.2 (0.5 to 2.8)	
Multi and grand multiparous (n’ = 174)	8.6	1	
**Patient abortion history**			**0.29**
One or several abortions (n’ = 146)	11.6	1.4 (0.7 to 2.7)	
No abortion history (n’ = 283)	8.5	1	

OR = Odds Ratio; P = p-value; F = Fisher's exact test

In this study, the median duration of hospitalization was 9 days, but it was significantly longer for patients who underwent surgery for pelviperitonitis due to uterine perforation (p<0.001) compared with the group of patients who underwent Caesarean section/hysterectomy. Moreover, the median duration of hospitalization was also significantly longer for the group of patients who were treated for other induced-abortion related complications (p<0.001) compared with patients treated for spontaneous abortion (Table 4).

**Table 4:** Duration of hospitalization for surviving women and analysis of variance according to the presence of induced abortion-related complications.

Median duration of hospitalization (P25-P75) = 9 (2–12) days			
Variables	N	Median duration (P25-P75) of hospitalization (days)	p
**Cases requiring surgery through the abdomen**			**< 0.001**
Postabortion pelviperitonitis	8	20 (4–36)	
Other cases of surgery through abdomen	587	10 (7–13)	
**Cases not requiring surgery through the abdomen**			**< 0.001**
Other complications of induced abortion	116	1 (1–3)	
Spontaneous abortions	128	0 (0–1)	

### Death characteristics

Of the 124 patients admitted to the hospital due to induced abortion-related complications, 5.6% (95% CI: 2.3–11.3%) died, and 42.9% of the deaths occurred after two days of hospitalization.

Furthermore, the researchers found that there was an increased risk of dying for patients who had postabortion pelviperitonitis (p<0.001) compared with other gynecological and obstetric emergencies (Tables 5 and 6).

**Table 5:** Frequency of deaths related to complications of induced abortions and deaths that occurred after two days of hospitalization.

Patients admitted because of induced abortion-related complication n = 124	n’ (%)	CI 95%
**Deaths**	** **	** **
Yes	7 (5.6)	2.3 to 11.3
No	117 (94.4)	87.7 to 97.7
Total	124 (100)	** **
Patients admitted who died because of induced abortion-related complication n = 7	n’ (%)	CI 95%
**Deaths after two days of hospitalization**		
Yes	3 (42.9)	15.8 to 75.0
No	4 (57.1)	25.0 to 84.2
Total	7 (100)	

**Table 6:** Frequency and characteristics of deaths related to complications of induced abortions.

Variables	% Deaths	OR (CI95%)	P
**Types of complications of induced abortion**	** **	** **	**0.06**
Postabortion pelviperitonitis (n’ = 8)	25.0	7.2 (0.6 to 57.3)	
Others (infection, hemorrhage) (n’ = 116)	4.3	1	
**Types of cases requiring surgery through the abdomen**	** **	** **	**< 0.001**
Postabortion pelviperitonitis (n’ = 8)	25.0	14.5 (1.3 to 91.7)	
Other cases of surgeries through abdomen (n’ = 586)	2.2	1	

## Discussion

In our study, 14.7% of the patients admitted to the obstetric and gynecological emergency units of the referral health facilities in Kinshasa had complications due to induced abortion. The findings of this study are similar to results reported in Nigerian studies on urban areas [11, 12]. In contrast, this diagnostic in rural health facilities appeared to be relatively infrequent compared with those reported in urban areas. For example, the complications due to induced abortion diagnosed in a tertiary hospital located in the Niger Delta [13] and a large rural hospital located in the southwest of Nigeria [14] were 5.6% and 7.4%, respectively. Similar results were also reported in three sub-Saharan countries [15].

We found a significant correlation between adolescence and the incidence of induced abortion-related complications. This finding is consistent with results found in many other studies carried out in comparable countries [16, 12, 17, 14, 11, 18, 19, 20].

A significant correlation with single, separated, divorced, or widowed women was also observed in the present study, and this result corroborates those reported by many other authors [12, 17, 14, 11, 21, 22, 23]. These results differ from those of some authors who found a significant correlation with marriage or cohabitation, including the results of Dragoman’s 2014 secondary analysis of data from the World Health Organization (WHO), which may be correlated to the probable confusion that would be induced by cases of complications resulting from spontaneous abortions that had not been discriminated against in the database [16]; the results of Igberase at a hospital located in rural Nigeria [13], which may reflect possible rural realities; and the results of Shah at a University Hospital in Pakistan and Erfani in Tehran, Iran [24, 25].

Furthermore, postabortion pelviperitonitis was found to be the most serious and urgent complication and accounted for 6.5% of all of the complications reported. Hemorrhage and infection were the most common complications, as was found in other studies [24, 26].

A greater number of abortion-related complications were associated with patients who resided in the semirural districts of Kinshasa. This may be due to the low socioeconomic level of the population, which could limit the purchase of medicines for postabortion care by women who have undergone abortion. Furthermore, a low socioeconomic level contributes to unsafe abortion practices.

Concerning the duration of hospitalization, the median length of stay was 9 days for all surviving patients. However, a significantly extended hospitalization was observed for patients who had undergone laparotomies for postabortion pelviperitonitis compared with patients who had undergone Caesarean sections/hysterectomies. The duration of hospitalization was also significantly longer for patients with complications resulting from induced abortion, having undergone vaginal intervention (aspiration, curetting, etc.) compared with the group of patients who had undergone spontaneous abortion. These results indicate that patients experiencing induced abortion-related complications have significantly longer hospital stays than patients with other comparable obstetric and gynecological complications, and that, in the DRC, this is indicative of a greater cost of supportive care for the former group of patients.

In this study, the mortality rate due to induced abortion complications was 5.6%. Thus, induced abortion-related complications are a significant problem in the DRC, and this observation is consistent with the results of many other studies that have been conducted in developing countries [12, 27, 17, 14, 28, 24, 29, 13, 30, 31, 32, 33]. It should also be noted that the lowest rates of mortality due to induced abortions are observed in countries where postabortion care units have been established [34, 35, 36, 37, 38, 39, 40].

Regarding the characteristics of death, we found an increased risk of death for patients with postabortion pelviperitonitis compared with other gynecological and obstetric emergencies. These results reflect the poor quality of care that women with post-abortion complications receive compared to other gynecological and obstetric conditions, as the mortality rates are lower under the same operating conditions. It is possible that the deficiencies in supportive care include either inappropriate postoperative treatments or discrimination against these patients by medical staff members due to their limited understanding of induced abortions. We also found that 42.9% of deaths occurred after two days of hospitalization. This latter finding supports our suspicion of insufficient supportive care for patients receiving aftercare for induced abortions, given that, conventionally, causes of death of patients who are admitted to hospital emergency units in the DRC are evaluated at the health facility when deaths occur after two days of hospitalization, while those that occur earlier (within 1–2 days) can be attributable to a cause upstream of the health facility.

It should be noted that this study probably underestimated the proportion of patients admitted for complications caused by induced abortions, as the diagnoses of induced or spontaneous abortions were guided by patients’ self-reports; generally, women are less likely to honestly report an induced abortion in settings where laws are restrictive and society is stigmatizing [41, 42, 43].

It should also be noted that, due to the lack of sufficient information in the records of patients who died for reasons other than induced abortions, it was very difficult for us to estimate the maternal mortality rate in the health facilities.

## Conclusion

The results of this study demonstrate that induced abortion-related complications represent major public health problems because of their frequencies among patients admitted to referral health facilities in Kinshasa, poor medical management, and the deaths related to them. Hospital-based supportive care received by patients did not prevent nearly half of the deaths from occurring after two days of hospitalization; deaths that occur in this time period are conventionally attributable to the health care system. Therefore, there is a need to understand the reason for poor medical management of these complications in order implement an adequate intervention program.

## Supporting information

S1 Database Database for five health facilities. (XLSX)

S1 File Ethics approval. (PDF)

## Abbreviations

DRC: Democratic Republic of the Congo
CI: confidence interval
SPSS: Statistical Package for the Social Sciences
OR: Odds Ratio
n: number of subjects in the sample
n’: number of subjects in the sample subgroups
SD: Standard Deviation
WHO: World Health Organization
p: p-value
F: Fisher's exact test
