# Supplementary material for: Analysis of induced abortion-related complications in women admitted to referral health facilities in Kinshasa, Democratic Republic of the Congo
Source: PLoS One. 2018 Aug 30;13(8):e0203186. doi: 10.1371/journal.pone.0203186 (PMC6117020; doi:10.1371/journal.pone.0203186)
Supplement: S1 File — (PDF) [file pone.0203186.s002.pdf]

REPUBLIQUE DEMOCRATIQUE DU CONGO
Ministère de l'Enseignement Supérieur, Universitaire et Recherche Scientifique
Université de Kinshasa
ECOLE DE SANTE PUBLIQUE
COMITE D'ETHIQUE

No d'Approbation: ESP/CE/052/14.

Kinshasa, le 19 décembre 2014

Au Dr Ishoso Katuashi Daniel
Investigateur Principal
Université de Kinshasa
Faculté deMédecine
Ecole Santé Publique

Concerne : Avis favorable concernant l'étude : *« Les complications des avortements provoqués admises aux établissements sanitaires de Kinshasa : ampleur, temps d'attente du traitement et issues».*

Docteur,

Le Comité d'Ethique de l'Ecole de Santé Publique de l'Université de Kinshasa a bien reçu le protocole dont le titre est repris en marge.

Après examen du protocole selon les normes d'éthique nationales sur les études impliquant les êtres humains, le Comité a donné un avis favorable à cette recherche et autorise sa mise en œuvre pour la période allant du 19 décembre 2014 au 18 décembre 2015.

Veuillez agréer, Docteur, l'expression de notre considération distinguée.

Prof. BONGOPASI MOKE SANGOL

Vice Président du Comité Ethique

ESP UNIKIN
COMITE D'ETHIQUE
